# Ventriculo-arterial coupling in acute heart failure: prognostic and therapeutic insights - a systematic review and meta-analysis of multivariable-adjusted data

**DOI:** 10.1007/s10741-026-10680-w

**Published:** 2026-10-06

**Authors:** Dragos Guz, Daniela Filipescu, Beth Davison, Elena-Laura Antohi, Andrew P. Ambrosy, Serban Mihaileanu, Oliviana Geavlete, Sean P. Collins, Razvan Radu, Marianna Adamo, Nicolas Girerd, Radu Filipescu, Luca Monzo, Jan Biegus, Gad Cotter, Ovidiu Chioncel

**Affiliations:** 1 University of Medicine and Pharmacy Carol Davila, 8 Eroii Sanitari Blvd, Bucharest, 050474 Romania; 2 Emergency Institute for Cardiovascular Diseases “Prof. Dr. C.C. Iliescu”, 258 Fundeni St, Bucharest, 022328 Romania; 3 Université Paris Cité, INSERM UMR-S 942 (MASCOT), Paris, France; 4 Heart Initiative, Momentum Research, Durham, NC USA; 5 Department of Cardiology, Division of Research, Kaiser Permanente San Francisco Medical Center, San Francisco, CA USA; 6 Department of Health Systems Science, Kaiser Permanente Bernard J. Tyson School of Medicine, Pasadena, CA USA; 7 Kaiser Permanente Northern California, Oakland, CA USA; 8 Institut Montsouris, Paris, France; 9 Department of Emergency Medicine, Vanderbilt University Medical Center, and Geriatric Research, Education and Clinical Care, Tennessee Valley Healthcare Facility VA Medical Center, Nashville, TN USA; 10 Department of Medical and Surgical Specialties, Radiological Sciences and Public Health, Institute of Cardiology, ASST Spedali Civili, University of Brescia, Brescia, Italy; 11 Centre d’Investigations Cliniques Plurithématique 1433, INSERM U1116 - DCAC, CHRU Nancy, F-CRIN INI-CRCT (Cardiovascular and Renal Clinical Trialists), Université de Lorraine, Nancy, France; 12 Department of Cardiology, Clinical Department of Intensive Cardiac Care, Institute of Heart Diseases, Wroclaw, Poland; 13 Faculty of Medicine, Wroclaw Medical University, Wroclaw, Poland

**Keywords:** Ventriculo-arterial coupling, Acute heart failure, Prognosis

## Abstract

In acute heart failure (AHF), non-invasive ventricular-arterial coupling (VAC) surrogates, including right ventricular-pulmonary arterial (RV-PA) and left ventricular-arterial (LV-Ao) coupling, have emerged as practical bedside indices, but their prognostic significance remains largely unknown systematically. We performed a systematic review and meta-analysis to evaluate the prognostic significance of non-invasive VAC assessment, in patients hospitalized with AHF. Outcomes included all-cause mortality, HF hospitalization, length of hospital stay, use of therapies, the composite of all-cause mortality and HF hospitalization, and in-hospital adverse cardiovascular events. Adjusted hazard ratios (aHRs) and odds ratios (aORs) were pooled using random-effects. Subgroup-analyses were performed according to follow-up duration and left ventricular ejection fraction. Thirty-six studies comprising 36,989 patients were analysed. Twenty-eight studies evaluated RV-PA coupling, predominantly the TAPSE/PASP ratio, and eight evaluated LV-Ao coupling, mainly using non-invasive Ea/Ees estimates. RV-PA uncoupling was independently associated with increased all-cause mortality (aHR 1.90; 95% CI 1.42–2.56; I²=49%), with consistent associations at 12 months (aHR 2.03; 95% CI 1.44–2.85) and long-term follow-up (aHR 1.67; 95% CI 1.06–2.64). Associations with HF hospitalization, hospital stay, and the composite endpoint were directionally consistent but not statistically significant. LV-Ao uncoupling was associated with increased in-hospital adverse cardiovascular events (aOR 1.70; 95% CI 1.29–2.24). Sensitivity analyses confirmed robustness of these findings. RV-PA uncoupling predicts mortality, whereas LV-Ao uncoupling identifies patients at increased risk of in-hospital adverse cardiovascular events, supporting VAC-risk-stratification in AHF and future evaluation of VAC-guided therapeutic strategies.

**Supplementary Information:**

The online version contains supplementary material available at https://doi.org/10.1007/s10741-026-10680-w.

## Introduction

Acute heart failure (AHF), defined as the new onset or worsening of signs and symptoms of heart failure (HF), remains a leading cause of unplanned hospital admission among patients older than 65 years [1–3]. Despite advances in therapy, AHF is associated with substantial morbidity and mortality [4].

The cardiovascular response to acute illness reflects the complex interaction between the heart and blood vessels, commonly referred to as ventriculo-arterial coupling (VAC). VAC involves matching ventricular contractility to afterload. Traditionally, this interaction has been characterized invasively using serial pressure-volume loop analyses obtained with conductance catheters [5]. Studies using this approach have substantially advanced our understanding of ventriculo-arterial interactions in both the left ventricle-systemic circulation (LV-Ao) [6–10] and the right ventricle-pulmonary arterial circulation (RV-PA) [11, 12]. VAC is quantified by the ratio of arterial elastance (Ea) to end-systolic elastance (Ees). Ea is a lumped parameter representing arterial load, whereas Ees provides a measure of LV contractile function and systolic stiffening.

In AHF, direct assessment of VAC is rarely feasible due to the practical limitations of manipulating loading conditions in hemodynamically unstable patients. Consequently, non-invasive surrogate measures of LV-Ao [13] and RV-PA coupling [14] are used in clinical practice and show good correlations with invasive measurements [13, 15]. Moreover, non-invasive echocardiographic-based hemodynamic indices have emerged as valuable bedside tools for prognostication in critically ill cardiovascular patients [16–21]. Although LV ejection fraction (LVEF) remains the most widely used parameter to assess cardiac function, it provides only limited assessment of cardiovascular performance, highlighting the need for a more comprehensive hemodynamic evaluation [22, 23].

To our knowledge, no prior systematic reviews have examined the association between non-invasive measures of VAC and clinical outcomes in patients hospitalized with AHF. We sought to determine the association between VAC and adverse outcomes in AHF, focusing on RV-PA coupling, assessed mainly by the tricuspid annular plane systolic excursion to estimated pulmonary artery systolic pressure (TAPSE/PASP ratio), and LV-Ao coupling, assessed by non-invasive estimates of Ea/Ees.

## Methods

This systematic review and meta-analysis was performed in accordance with the Cochrane Collaboration Handbook for Systematic Review of Interventions and the Preferred Reporting Items for Systematic Reviews and Meta-Analysis (PRISMA) Statement guidelines [24, 25].

### Eligibility criteria

Inclusion criteria for this meta-analysis were restricted to studies that met all the following eligibility criteria: (1) randomized or non-randomized cohort studies, including prospective or retrospective designs; (2) enrolling adult patients with AHF; (3) assessing VAC using invasive or non-invasive methods; (4) comparing AHF patients with non-AHF subjects or within VAC strata; and (5) reporting at least one outcome of interest. No restrictions were applied regarding follow-up duration. Studies were excluded if they were (1) non-original; (2) non-human; or (3) did not allow extraction of AHF-specific data.

### Search strategy and data

We systematically searched PubMed, Web of Science, and Scopus from inception to December 2025 using the following search terms: ‘acute heart failure’, ‘ventriculo-arterial coupling’, ‘arterial elastance’, ‘end-systolic elastance’, ‘pressure-volume analysis’, and ‘myocardial work’. We also manually searched the references of all included studies, previous systematic reviews, and meta-analyses for additional studies. Two authors (D.G. and R.F.) independently extracted data according to predefined search criteria and quality assessment. One additional study [26], published after completion of the systematic search, was identified during the revision process and incorporated into the synthesis.

### Endpoints and subgroup analyses

Outcomes of interest included all-cause mortality, HF hospitalization, length of hospital stay, use of therapies, and in-hospital adverse cardiovascular events - defined at the study level as a composite of in-hospital death and/or the need for invasive ventilation, intra-aortic balloon pump, or renal replacement therapy. We also assessed a composite outcome of all-cause mortality and HF hospitalization. We aimed to perform subgroup analyses for (1) all-cause mortality by duration of follow-up (12-month and long-term) and (2) all-cause mortality across the LVEF spectrum.

### Quality assessment

Non-randomized studies were appraised using the Risk of Bias in Non-randomized Studies - of Interventions tool (ROBINS-I) [27]. Quality assessment of randomized controlled trials was performed using version 2 of the Cochrane Risk of Bias assessment tool (Supplemental Table 1) [28]. Two independent authors completed the risk of bias assessment (D.G. and R.F.). Disagreements were resolved through consensus after discussion of the reasons for discrepancy. Publication bias was investigated using a funnel-plot analysis of point estimates in relation to study weight (Supplemental Fig. 4).

### Statistical analysis

Pooled adjusted hazard ratios (aHRs) with 95% confidence intervals (CIs) were used for time-to-event outcomes. For binary endpoints, including in-hospital adverse cardiovascular events, effect estimates were synthesized as adjusted odds ratios (aORs). Adjusted effect measures were log-transformed and pooled using inverse-variance weighting. A two-sided p-value < 0.05 was considered statistically significant for pooled effect estimates. We applied a random-effects model with between-study variance (τ²) estimated using restricted maximum likelihood (REML). To account for uncertainty in τ² estimation, we additionally applied the Hartung-Knapp-Sidik-Jonkman (HKSJ) method, or the Wald-type method when appropriate, to provide more robust confidence intervals in the random-effects framework. Statistical heterogeneity was assessed using Cochran’s Q test and quantified with the I² statistic; p-values < 0.10 and I² >25% were considered suggestive of heterogeneity. Differences between the predefined subgroups (follow-up duration and LVEF phenotype) were evaluated using the χ²-based test for subgroup differences derived from Cochran’s Q statistic, which partitions the overall heterogeneity into between- and within-subgroup components; the corresponding p-value for subgroup differences is reported for each stratified analysis. We also performed an exploratory random-effects meta-regression of all-cause mortality on the study-level TAPSE/PASP cut-off (REML, Knapp-Hartung), summarizing the heterogeneity explained as a pseudo-R². Statistical analyses were performed using Review Manager 7.2.0 (Cochrane Center, The Cochrane Collaboration, Denmark) and R version 4.5.1 (R Core Team, R Foundation for Statistical Computing, Vienna, Austria).

## Results

### Study selection and baseline characteristics

**Fig. 1:**
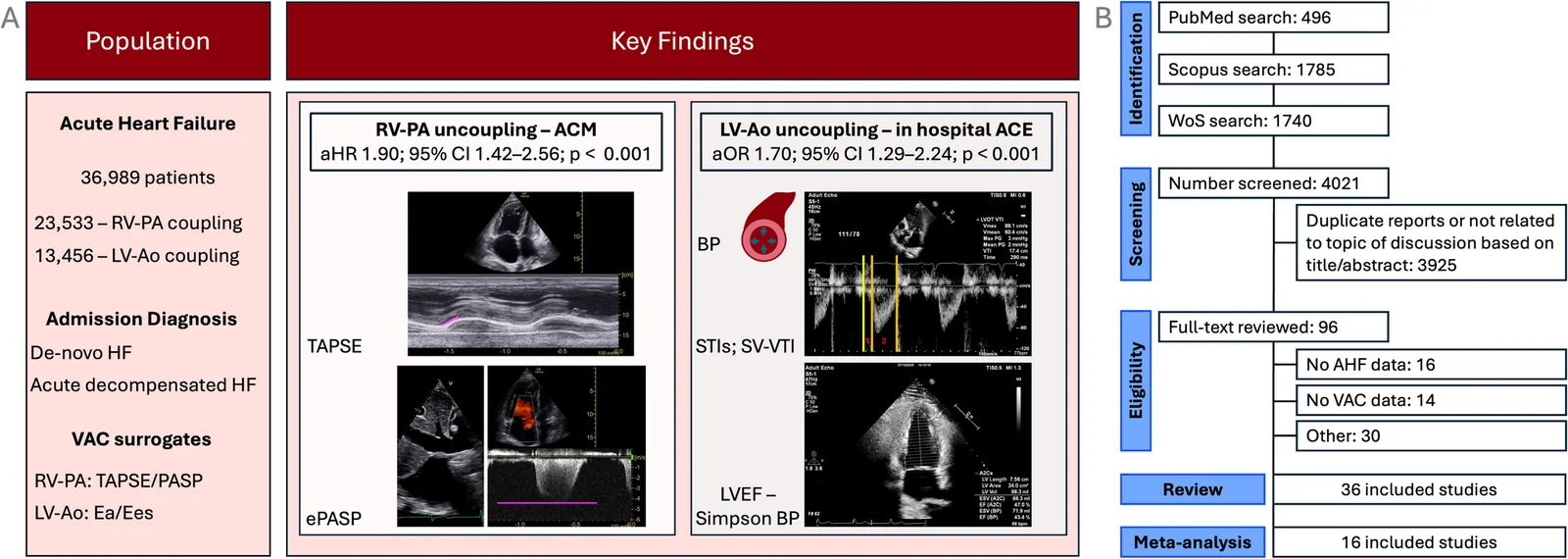
**(A)** Graphical abstract: Population and main findings. 36,989 patients admitted with de-novo or acutely decompensated AHF, characterized by ventricular-arterial coupling: bedside non-invasive hemodynamic parameter representative of the right ventricle, respectively left ventricle and afterload interplay. **(B)** PRISMA flow diagram of study screening and selection Abbreviations: ACE = adverse cardiovascular events; ACM = all-cause mortality; AHF = acute heart failure; aHR = adjusted hazard ratio; aOR = adjusted odds ratio; BP = blood pressure; CI = confidence interval; ePASP = estimated pulmonary artery systolic pressure (estimated right atrial pressure plus right ventricle–right atrium pressure gradient); HF = heart failure; LV-Ao = left ventricle–systemic circulation; LVEF Simpson BP = left ventricular ejection fraction measured by the Simpson biplane method; RV-PA = right ventricle–pulmonary circulation; STIs = systolic time intervals; SV–VTI = stroke volume calculated using left ventricular outflow tract diameter and velocity–time integral; TAPSE = tricuspid annular plane systolic excursion; VAC = ventriculo–arterial coupling; WoS = Web of Science

The initial search yielded 4021 results. After removing duplicate records and ineligible studies, 96 remained and were fully reviewed against the inclusion criteria. Ultimately, 36 studies were included in the systematic review, comprising 36,989 patients from 1 randomized controlled trial and 35 non-randomized cohorts (15 prospective and 20 retrospective; Tables 1 and 2; Fig. 1B).

**Table 1:** Baseline characteristics of included studies evaluating RV-PA coupling surrogates. DM – diabetes mellitus; E-PA=RVSP/SV: effective pulmonary arterial elastance, calculated as the ratio of right ventricular systolic pressure to stroke volume; IHD – ischemic heart disease; LVEF – left ventricular ejection fraction; NA - not available; RV-fwLS/PASP – ratio of right ventricular free-wall longitudinal strain to pulmonary artery systolic pressure; RVGLS/PASP- ratio of right ventricular global longitudinal strain to pulmonary artery systolic pressure; RVSP/SBP: ratio of right ventricular systolic pressure to systemic systolic blood pressure; TAPSE/PASP - ratio of tricuspid annular plane systolic excursion to pulmonary artery systolic pressure; TASV/PASP - ratio of tricuspid annular systolic velocity to pulmonary artery systolic pressure; TAPSE/TRV - ratio of tricuspid annular plane systolic excursion to tricuspid regurgitation velocity; TTE – transthoracic echocardiography; VAC – ventriculo-arterial coupling. Age reported as mean ± standard deviation or median [IQR]; † included in the quantitative synthesis

Study	Study design	Center	No. of patients	LVEF	Female patients	Age	IHD	DM	VAC surrogate	VAC cut-off	Timing of VAC measurement	Follow-up
Nakagawa et al.,[32]^†^	Prospective cohort	32; JPN	655	≥ 50%	56%	83 [77–87]	14%	32%	TTE: TAPSE/PASP	0.481 mm/mmHg	at discharge	12 months
Palazzuoli et al.,[40]^†^	Prospective cohort	1; EU	381	< 40%, 40–49%, ≥ 50%	53%	82 [77–86]	37.30%	48%	TTE: TAPSE/PASP	0.425 mm/mmHg	≤ 12 h admission	20.8 months
Santas et al.,[33]^†+^	Prospective cohort	1; EU	884	≥ 50%	64.40%	76.1 ± 9.7	20.90%	39.20%	TTE: TAPSE/PASP	0.36 mm/mmHg	index hospitalization	12 months
Santas et al.,[77]	Retrospective cohort	1; EU	1948	< 40%, 40–49%, ≥ 50%	49.30%	74 ± 11	29.20%	43.10%	TTE: TAPSE/PASP	0.36 mm/mmHg	index hospitalization	18 months
Jentzer et al.,[29]^†^	Retrospective cohort	1; US	4259	< 40%, 40–49%, ≥ 50%	40.10%	69.3 ± 14.9	17.60%	NA	TTE: TASV/PASP	< 0.18 vs. ≥ 0.43 cm/sec/mmHg	≤ 24 h admission	12 months
Naseem et al.,[78]	Prospective cohort	1; EGY	200	< 40%	43.50%	56.36	51.30%	18.50%	TTE: TAPSE/PASP	0.4 mm/mmHg	at admission	3 months
Hullin et al.,[46]	Prospective cohort	1; EU	176	< 40%, 40–49%, ≥ 50%	46%	83 [74–87]	44%	37%	TTE: TAPSE/PASP	0.23 mm/mmHg	at admission &discharge	57 months
Berrill et al.,[79]	Prospective cohort	1; UK	418	< 40%, 40–49%, ≥ 50%	30.14%	62	56.90%	45%	TTE: TAPSE/PASP	0.27 mm/mmHg	≤ 24 h admission	35 months
Falsetti et al.,[30] ^†^	Retrospective cohort	1; EU	439	< 40%, 40–49%, ≥ 50%	59%	84.6 ± 7.7	NA	NA	TTE: TAPSE/PASP	0.325 mm/mmHg	≤ 24 h admission	CICU stay
Vella et al.,[80]	Prospective cohort	1; EU	110	< 40%, 40–49%, ≥ 50%	42%	73 ± 12	42%	39%	TTE: TAPSE/PASP	0.3 mm/mmHg	at admission	24 months
Palazzuoli et al.,[54]	Retrospective cohort	3; EU	380	< 40%, 40–49%, ≥ 50%	32.90%	81 [77–86]	54.50%	46.80%	TTE: TAPSE/PASP	0.49 mm/mmHg	at admission	6 months
Bok et al.,[36] ^†^	Retrospective cohort	1; KOR	1147	< 40%, 40–49%, ≥ 50%	49.87%	70.81 ± 12.56	34.40%	33.60%	TTE: TAPSE/PASP	0.33 mm/mmHg	index hospitalization	29 months
Bonelli et al.,[34] ^†^	Retrospective cohort	1; EU	340	< 40%, 40–49%, ≥ 50%	24%	68.8 ± 11.8	48%	35%	TTE: TAPSE/PASP	Quartiles (study-specific)	at admission	12 months
Jia et al.,[35] ^†^	Prospective cohort	1; CHN	250	≥ 50%	36.40%	66 [56–73]	100%	38.40%	TTE: TAPSE/PASP	0.43 mm/mmHg	index hospitalization	14 months
Bowcock et al.,[81]	Retrospective cohort	1; AUS	1077	< 40%, 40–49%, ≥ 50%	39%	69 [58, 77]	NA	NA	TTE: TAPSE/TRV	Quartiles (study-specific)	index hospitalization	in-hospital
Park et al.,[82]	Retrospective cohort	3; KOR	2865	< 40%, 40–49%, ≥ 50%	49.40%	71.1 ± 13.5	32.30%	34.70%	TTE: RVGLS/PASP	≤ 0.32%/mmHg	index hospitalization	12 months
Baldetti et al.,[83]	Retrospective cohort	2; EU	127	< 30%	39.40%	65 [52–73]	NA	NA	PAC: E-PA=PASP/SV	0.85 mmHg/mL	at admission &24 h later	24 months
Fauvel et al.,[67]	Retrospective cohort	39; EU	333	< 40%, 40–49%, ≥ 50%	29.80%	69 ± 14	25%	30%	TTE: TAPSE/PASP	0.4 mm/mmHg	≤ 24 h admission	in-hospital stay
Berrill et al.,[37] ^†^	Prospective cohort	1; UK	391	< 40%, 40–49%, ≥ 50%	47%	79	NA	NA	TTE: TAPSE/PASP	0.24 mm/mmHg	NA	26 months
Padkins et al.,[84]	Retrospective cohort	1; US	4939	< 40%, 40–49%, ≥ 50%	40.90%	72.2 [60.3–80.5]	18.20%	28.20%	TTE: E-PA=RVSP/SV; RVSP/SBP; TASV/PASP	Quartiles (study-specific)	≤ 24 h admission	12 months
Burgos et al.,[39] ^†^	Retrospective cohort	1; ARG	361	> 40%	51%	76 ± 13	NA	26.30%	TTE: TAPSE/PASP	0.38 mm/mmHg	index hospitalization	20 months
Palazzuoli et al.,[55]	Retrospective cohort	5; EU	425	< 40%, 40–49%, ≥ 50%	54.82%	79.4 ± 9.5	44%	50%	TTE: TAPSE/PASP, RV-fwLS/PASP	0.38 mm/mmHg; 0.48%/mmHg	at admission	6 months
Antit et al., [38]^†^	Prospective cohort	1; TUN	124	< 40%, 40–49%, ≥ 50%	30.46%	65 ± 10	50%	66%	TTE: TAPSE/PASP	0.35 mm/mmHg	at admission	18 months
De Matteis et al.,[31] ^†^	Retrospective cohort	1; EU	398	≥ 50%	56%	83 [75–87]	24.10%	23.10%	TTE: TAPSE/PASP	0.36 mm/mmHg	index hospitalization	12 months
Kosmidis et al.,[44]	Prospective cohort	1; EU	221	< 40%, 40–49%, ≥ 50%	59%	82	NA	NA	TTE: TAPSE/PASP	0.45 mm/mmHg	≤ 24 h admission	12 months
Decotto et al.,[41] ^†^	Prospective cohort	1; ARG	142	≥ 50%	65%	84 [78–88]	15.20%	20.40%	TTE: TAPSE/PASP	0.4 mm/mmHg	≤ 24 h admission	in-hospital
Husain-Syed et al.,[45]	Retrospective cohort	1; EU	53	< 40%, 40–49%, ≥ 50%	26.40%	73.2 ± 12.8	52.80%	41.50%	TTE: TAPSE/PASP	NA (continuous analysis)	≤ 24 h admission	60 months
Anastasiou et al.,[26] ^†^	Retrospective cohort	1; EU	490	< 40%, 40–49%, ≥ 50%	40.60%	74 ± 11.9	37.10%	36%	TTE: TAPSE/PASP	0.36 mm/mmHg	≤ 24 h admission; 24 h pre-discharge	12 months

**Table 2:** Baseline characteristics of included studies evaluating LV-Ao coupling surrogates. DM – diabetes mellitus; Ea – arterial elastance; Ees – ventricular elastance; ESV – end-systolic volume; IHD – ischemic heart disease; LVEF – left ventricular ejection fraction; LVSWI – left ventricular stroke work index; MW – myocardial work; NA - not available; SV – stroke volume; TTE – transthoracic echocardiography; VAC – ventriculo-arterial coupling. Age reported as mean ± standard deviation or median [IQR]. † included in the quantitative synthesis

Study	Study design	Center	No. of patients	LVEF	Female patients	Age	IHD	DM	VAC surrogate	VAC surrogate cut-off	Timing of VAC measurement		Follow-up
Berthelot et al.,[85]	Prospective cohort	1; EU	19	< 40%	0%	62 ± 15	42%	37%	TTE, invasive arterial pressure: Ea/Ees	NA	at admission	in-hospital	
Trambaiolo et al.,[47]	Retrospective cohort	1; EU	48	< 40%	8%	64 ± 12	40%	38%	TTE: Ea/Ees (Chen)	early ACM: VAC = 1.99; 12 months ACM: VAC = 1.68	at admission &discharge	12 months	
Jentzer et al.,[17]	Retrospective cohort	1; US	4536	< 40%, 40–49%, ≥ 50%	36%	68 ± 14.3	18.70%	28.10%	TTE: LVSWI	in hospital: LVSWI 31 g*min/m2; 12 months: LVSWI 20 g×min/m2 (adjusted)	≤ 24 h admission	12 months	
Sahiti et al.,[49]	Prospective cohort	1; EU	126	< 40%, 40–49%, ≥ 50%	37%	73 ± 12	25%	41%	TTE: ∆MW	GWW: 10 mmHg% increments	at admission &24 h after inotrope infusion	6 months	
Trambaiolo et al.,[43] ^†^	Prospective cohort	1; EU	329	< 40%, 40–49%, ≥ 50%	23.10%	66.7 ± 15.5	32.10%	32.80%	TTE: VAC = Ea/Ees (Chen)	NA	≤ 24 h admission	in-hospital	
Jentzer et al.,[18]	Retrospective cohort	1; US	3653	< 40%, 40–49%, ≥ 50%	37%	68.1 ± 14.5	61.10%	NA	TTE: LVSWI	33.1 J/m2	*≤ 24 h admission*	in-hospital	
Liang et al.,[48]	RCT	3; CHN	60	< 40%	43.30%	63 ± 12	100%	38.30%	TTE: ∆MW	NA	≤ 12 h admission &before discharge	6 months	
Fazzini et al.,[42]^†^	Retrospective cohort	1; US	4685	< 40%, 40–49%, ≥ 50%	34.40%	69 [58, 79]	NA	NA	TTE: Ea/Ees = ESV/SV	2	at discharge; after 1-, 2-, 3- &6-months	12 months	

Among these, 28 studies evaluating RV-PA coupling surrogates (*n* = 23,533; 63.6%) were identified, of which 14 studies (*n* = 10,261; 43.6%) were included in the meta-analysis (Table 1). Eight studies assessed LV-Ao coupling (*n* = 13,456; 36.4%); however, only two studies (*n* = 5,014; 37.3%) were eligible for quantitative synthesis (Table 2). Definitions and cut-off values used to characterize ventriculo-arterial uncoupling varied across studies.

RV-PA coupling was evaluated using non-invasive echocardiographic surrogates, most commonly the TAPSE/PASP ratio, with measurements obtained at admission or within 24 h of index hospitalization. Jentzer et al. defined coupling as the ratio of tricuspid annular peak systolic tissue Doppler velocity (TASV) to estimated PASP [29]. TASV and TAPSE both measure RV longitudinal systolic function, and the two ratios were strongly correlated (*r* = 0.88; *p* < 0.001) [29]. Follow-up ranged from in-hospital outcomes to 60 months. TAPSE/PASP cut-offs ranged from 0.23 to 0.48 mm/mmHg, most commonly 0.36–0.38 mm/mmHg in Western cohorts and up to 0.48 mm/mmHg in Asian cohorts. In studies assessing LV-Ao coupling, VAC was similarly evaluated non-invasively, most frequently using the Ea/Ees, with follow-up extending from in-hospital endpoints to 12 months (Table 2).

Across RV-PA coupling studies, the weighted mean age was 72.7 years (study-level range 56.4–84.6), 44.9% were women, and the prevalence of ischemic heart disease and diabetes mellitus was 27.2% and 34.6%, respectively. Five cohorts enrolled exclusively patients with AHF with preserved LVEF (AHFpEF), two enrolled only patients with AHF with reduced LVEF (AHFrEF), and 20 included patients across the EF spectrum; one study enrolled patients with LVEF > 40%. Most studies originated from Europe (*n* = 15), with the remainder from East Asia (*n* = 4), United States (*n* = 2), United Kingdom (*n* = 2), Middle East and North Africa (*n* = 2), South America (*n* = 2), and Australia (*n* = 1); of these, six were multicentre.

In LV-Ao coupling studies, patients were younger (weighted mean age 68.3 years; range 62.0–73.0), with a lower proportion of women (35.3%) and a higher prevalence of ischemic heart disease (37.7%). Three cohorts included only patients with AHFrEF, and five spanned the full EF spectrum.

### Pooled analyses of all included studies

#### RV-PA coupling and clinical outcomes

**Fig. 2:**
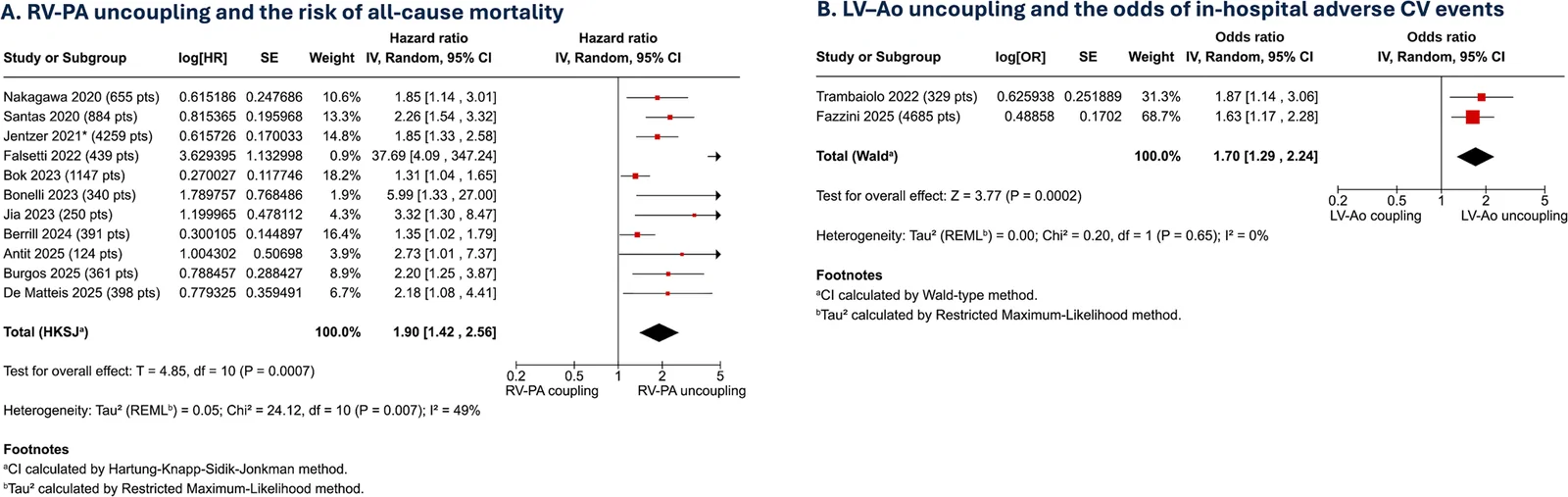
Ventriculo-arterial uncoupling and adverse clinical outcomes in AHF: **(A)** RV-PA uncoupling is associated with increased risk of all-cause mortality; **(B)** LV-Ao uncoupling is significantly associated with higher odds of in-hospital adverse cardiovascular events. Abbreviations: AHF = acute heart failure; LV-Ao = left ventricle–systemic circulation; RV-PA = right ventricle–pulmonary circulation. Jentzer 2021* effect estimate source: Figure 3B (Hazard ratios for TASV/RVSP quintiles were extracted from Figure 3B using WebPlotDigitizer (Automeris v5.2)). Comparator: Q1 vs Q5. In Jentzer et al., coupling was defined as tricuspid annular systolic velocity (TASV, S′) divided by estimated pulmonary artery systolic pressure, with TAPSE/PASP calculated when available. TASV and TAPSE were considered equivalent measures of right ventricular longitudinal systolic function. In the leave-one-out sensitivity analysis the results were not dependent on the Jentzer 2021 study.

RV-PA uncoupling, primarily assessed using TAPSE/PASP, was associated with a significantly increased risk of *all-cause mortality*, based on pooled multivariable-adjusted estimates from observational studies (aHR 1.90, 95% CI 1.42–2.56; *p* = 0.0007; I² = 49%; Fig. 2A) [29–39]. The association with mortality was consistent across studies despite variable thresholds; however, most studies used cut-offs around 0.36–0.38 mm/mmHg (Western cohorts).

**Fig. 3:**
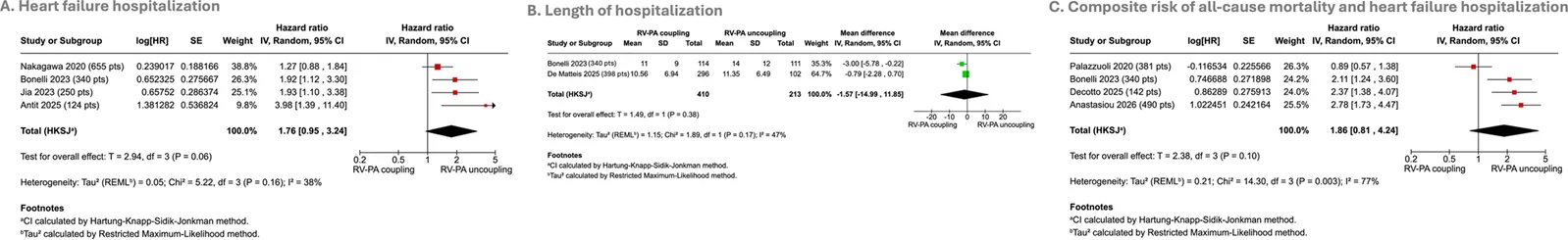
RV-PA uncoupling was associated with a trend toward: **A**. Increased heart failure hospitalization; **B**. Longer hospital stay; **C**. Increased composite risk of all-cause mortality and heart failure hospitalization Abbreviations: RV-PA = right ventricle–pulmonary circulation

For *HF hospitalization*, there was an overall trend toward increased risk in patients with RV-PA uncoupling (aHR 1.76, 95% CI 0.95–3.24; *p* = 0.06; I² = 38%; Fig. 3A) [32, 34, 35, 38]. Preserved RV-PA coupling (TAPSE/PASP > 0.36 mm/mmHg) was associated with a trend toward a shorter *length of hospital stay*; however, this did not reach statistical significance (mean difference − 1.57 days, 95% CI − 14.99 to 11.85; *p* = 0.38; I² = 47%; Fig. 3B) [31, 34]. The *composite outcome of all-cause mortality and HF hospitalization* showed a non-significant trend toward increased risk in patients with RV-PA uncoupling (TAPSE/PASP < 0.36 mm/mmHg; aHR 1.86, 95% CI 0.81–4.24; *p* = 0.10; I² = 77%; Fig. 3C) [26, 34, 40, 41].

#### LV-Ao coupling and in-hospital adverse cardiovascular events

LV-Ao uncoupling, most frequently assessed using Ea/Ees (> 2), was significantly associated with higher odds of in-hospital adverse cardiovascular events (in-hospital death and/or the need for intra-aortic balloon pump, invasive ventilation, or renal replacement therapy) in pooled analyses (aOR 1.70, 95% CI 1.29–2.24; *p* = 0.0002; I² = 0%; Fig. 2B), with no evidence of between-study heterogeneity. The endpoint was derived from two complementary designs: a large cardiac intensive care unit cohort [42], contributing in-hospital mortality, and an unselected AHF cohort [43], contributing a composite of mechanical circulatory, respiratory, or renal support and in-hospital death.

### Outcomes not eligible for meta-analysis

Data on *therapy use* were not suitable for quantitative synthesis. In a prospective cohort study by Kosmidis et al., inotropic therapy was used in 35% of patients [44]. RV-PA uncoupling, defined as TAPSE/PASP < 0.30 mm/mmHg, was associated with higher adjusted odds of inotrope use (aOR 2.6; 95% CI 1.3–5.2; *p* = 0.006) [44]. In a separate prospective cohort with serial coupling assessment, persistent RV-PA uncoupling at discharge was also associated with more frequent use of inotropic agents than restored coupling (17.9% vs. 8.8%; *p* = 0.033) [26].

In the study by Bonelli et al., prior to admission, patients with lower TAPSE/PASP values received higher diuretic doses [34]. During hospitalization, patients with RV-PA uncoupling at admission (TAPSE/PASP < 0.34 mm/mmHg) [34] and those with persistent uncoupling at discharge (TAPSE/PASP < 0.36 mm/mmHg) required higher doses of intravenous diuretics (197.7 ± 127.2 vs. 135.6 ± 108.7 mg/day; *p* < 0.001) [26] and were discharged on higher oral diuretic doses [26, 34].

In the study by Husain-Syed et al., increases in TAPSE/PASP after decongestion were associated with higher odds of discontinuing renal replacement therapy (OR 1.83, 95% CI 1.03–3.32; *p* = 0.049) [45]. In a separate cohort study by Hullin et al., a higher TAPSE/PASP after decongestion was associated with a lower incidence of the composite outcome of all-cause mortality and cardiovascular rehospitalization at 3 months; however, this association was observed only in univariable analysis (OR 0.29, 95% CI 0.001–0.55; *p* = 0.02) [46]. Normalized RV-PA coupling after AHF treatment was associated with 12-month event-free survival comparable to that of patients with preserved coupling (TAPSE/PASP ≥ 0.36 mm/mmHg) on admission (71.5% vs. 74.1%; *p* < 0.001) [26].

In the study by Trambaiolo et al., baseline LV-Ao uncoupling, assessed using Ea/Ees (Chen method), predicted the need for escalation to combined vasopressor and inotropic therapy in patients with ST-segment elevation myocardial infarction complicated by de novo AHF [47]. LV-Ao uncoupling was also associated with both early all-cause mortality (Ea/Ees ≥ 1.99; AUC 0.786; sensitivity 67%; specificity 82%) and long-term all-cause mortality (Ea/Ees ≥ 1.68; AUC 0.769; sensitivity 66%; specificity 67%) [47]. In a separate study evaluating myocardial work parameters derived from LV pressure-strain loop analysis, baseline Global Constructive Work emerged as an independent predictor of major cardiovascular events after multivariable adjustment (aOR 1.08; *p* < 0.001; AUC 0.78; *p* < 0.05) [48]. In another cohort of 126 patients with AHFrEF, higher Global Wasted Work was associated with increased risk of death or rehospitalization within 6 months after discharge (per 10-point increment, HR 1.035, 95% CI 1.005–1.065) [49].

Among critically ill intensive care unit patients, 10.6% had AHF. LV-Ao uncoupling was an independent predictor of a composite outcome that included the need for intra-aortic balloon pump support, orotracheal intubation, or continuous renal replacement therapy, even after adjustment for comorbidities (aOR 1.87, 95% CI 1.14–3.06; *p* = 0.013) [43].

### Subgroup analyses

**Fig. 4:**
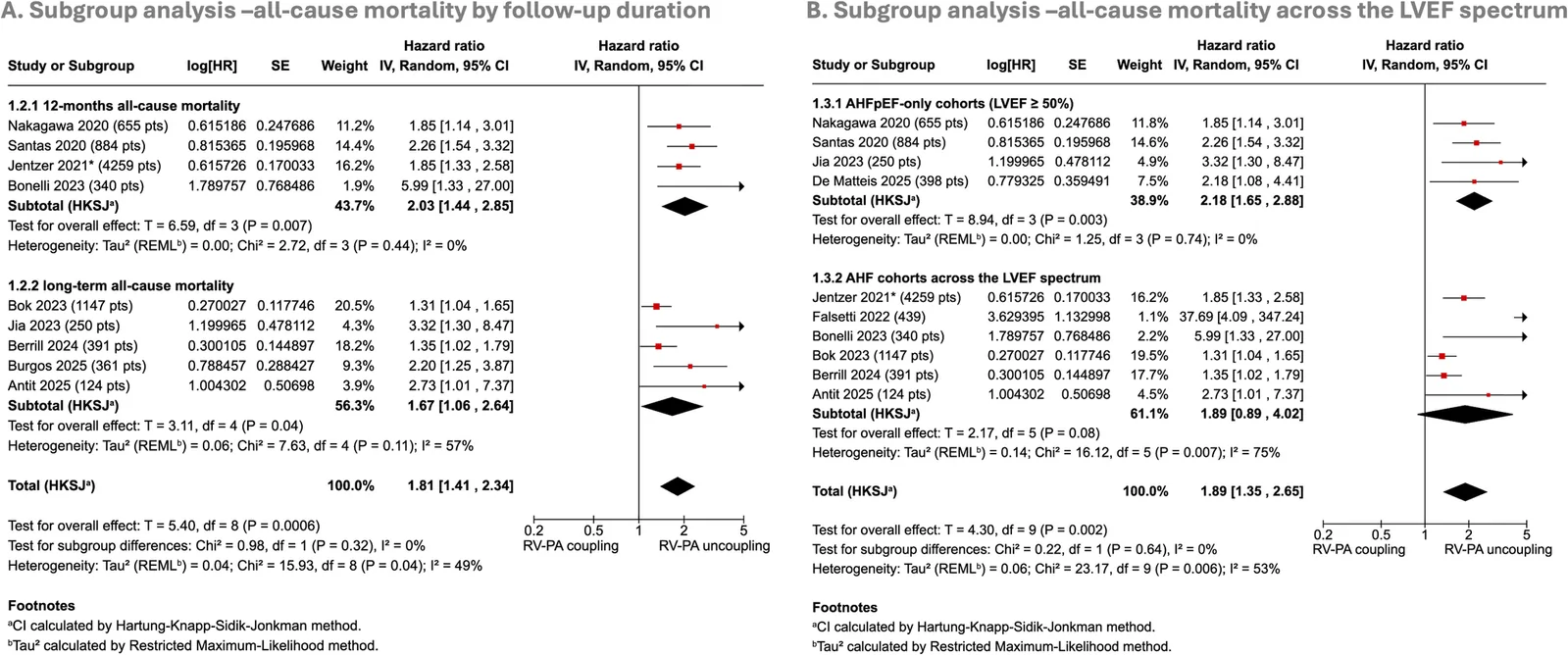
**(A)** Subgroup analyses stratified by follow-up duration: RV-PA uncoupling remained associated with an increased risk of all-cause mortality; **(B)** Subgroup analyses for all-cause mortality across the LVEF spectrum: RV-PA uncoupling was significantly associated with increased all-cause mortality in AHFpEF-only; in contrast, among AHF cohorts spanning the LVEF spectrum, RV-PA uncoupling was associated with a non-significant trend toward higher all-cause mortality. Abbreviations: AHFpEF = acute heart failure with preserved ejection fraction; LVEF = left ventricular ejection fraction; RV-PA = right ventricle–pulmonary circulation. Jentzer 2021* effect estimate source: Figure 3B (Hazard ratios for TASV/RVSP quintiles were extracted from Figure 3B using WebPlotDigitizer (Automeris v5.2)). Comparator: Q1 vs Q5. In Jentzer et al., coupling was defined as tricuspid annular systolic velocity (TASV, S′) divided by estimated pulmonary artery systolic pressure, with TAPSE/PASP calculated when available. TASV and TAPSE were considered equivalent measures of right ventricular longitudinal systolic function. In the leave-one-out sensitivity analysis the results were not dependent on the Jentzer 2021 study

In subgroup analyses stratified by *follow-up duration (12 months vs. long-term)* RV-PA uncoupling remained associated with an increased risk of all-cause mortality. At 12 months, pooled analysis demonstrated a significantly higher mortality risk in patients with RV-PA uncoupling (aHR 2.03; 95% CI 1.44–2.85; *p* = 0.007; I² = 0%; Fig. 4A), with no evidence of between-study heterogeneity [29, 32–34]. Similarly, long-term follow-up analyses showed a significant association between RV-PA uncoupling and all-cause mortality (aHR 1.67; 95% CI 1.06–2.64; *p* = 0.04; I^2^ = 56%; Fig. 4A) [35–39]. The association between RV-PA uncoupling and all-cause mortality did not differ according to follow-up duration (p for interaction = 0.32).

In subgroup analyses for all-cause mortality *across the LVEF spectrum*, cohorts were classified as AHFpEF-restricted or full-spectrum. In AHFpEF-restricted cohorts, RV-PA uncoupling was significantly associated with increased all-cause mortality (aHR 2.18; 95% CI 1.65–2.88; *p* = 0.003; I² = 0%; Fig. 4B), without between-study heterogeneity [31–33, 35]. By comparison, across cohorts spanning the full LVEF spectrum (excluding AHFpEF cohorts only), RV-PA uncoupling (TAPSE/PASP < 0.36 mm/mmHg) was not significantly associated with all-cause mortality and the heterogeneity was high (aHR 1.89; 95% CI 0.89–4.02; *p* = 0.08; I² = 75%; Fig. 4B) [29, 30, 34, 36–38]. However, the test for subgroup differences was not statistically significant (*p* = 0.64).

### Sensitivity analyses

Due to high heterogeneity, we performed a “leave-one-out sensitivity analysis” by iteratively removing one study at a time to ensure the results were not dependent on a single study. Overall, removing each study from the pooled analysis did not affect the endpoint of *all-cause mortality* (Supplemental Fig. 1A). By contrast, for *HF hospitalization*, excluding the Nakagawa study [32] rendered the pooled association statistically significant (aHR 2.10; 95% CI 1.46–3.03; *p* < 0.0001; I^2^ = 0%; Supplemental Fig. 1B). This was attributable to the substantial weight of the Nakagawa cohort [32], which reported an attenuated, non-significant adjusted effect estimate in an Asian AHFpEF-only population. For the *composite outcome of all-cause mortality and HF hospitalization*, restricting the analysis to studies reporting 12-month follow-up, thereby excluding the only 6-month cohort [40], rendered the pooled association statistically significant (aHR 2.44; 95% CI 1.72–3.46; *p* = 0.008; I² = 0%; Supplemental Fig. 1C). Similarly, in analyses of RV-PA uncoupling across the *LVEF spectrum*, excluding the Falsetti study [30] reduced heterogeneity and yielded a statistically significant association with *all-cause mortality* (aHR 1.55; 95% CI 1.05–2.28; *p* = 0.04; I² = 36%; Supplemental Fig. 2), driven by an imprecise effect estimate with a very wide confidence interval.

Sensitivity analyses restricted to studies using TAPSE/PASP cut-offs of 0.36–0.38 mm/mmHg yielded consistent results, confirming the robustness of the primary analysis and preserving both the direction and magnitude of the effect (aHR 2.23; 95% CI 1.67–2.98; *p* < 0.00001; I² = 0%; Supplemental Fig. 3).

### Quality assessment

For quantitative analyses, only multivariable-adjusted risk estimates were included. The detailed risk-of-bias appraisal is summarized in Supplemental Table 1. Funnel plot analysis of the primary outcome (all-cause mortality) demonstrated a symmetrical distribution of studies for the 12-month follow-up subgroup (Supplemental Fig. 4A). In contrast, when considering all available follow-up periods combined (ranging from in-hospital to long-term all-cause mortality), the funnel plot appeared asymmetrical (Supplemental Fig. 4B), indicating potential small-study effects.

## Discussion

In this systematic review and meta-analysis of 36 studies including 36,989 patients with AHF, we evaluated the prognostic significance of the right- and left-sided VAC-surrogates using multivariable-adjusted estimates. Among patients with RV-PA uncoupling, assessed predominantly by the TAPSE/PASP ratio, three key findings emerged: (1) a significantly higher risk of all-cause mortality, which remained consistent across medium- and long-term follow-up; (2) a trend toward increased HF hospitalization and a longer hospital stay; and (3) a non-significant increase in the composite endpoint of all-cause mortality and HF hospitalization. In contrast, among patients with LV-Ao uncoupling, evaluated predominantly by non-invasive estimates of Ea/Ees ratio, the odds of in-hospital adverse cardiovascular events were significantly higher.

Collectively, these findings support the prognostic relevance of VAC surrogates even in AHF settings.

### RV-PA coupling and clinical outcomes in acute heart failure

The principal finding of the present meta-analysis is that RV-PA uncoupling is independently associated with *all-cause mortality* in patients hospitalized with AHF. These findings extend previous observations in chronic HF [50] to the acute setting and support the prognostic role of RV-PA coupling in AHF. The stronger association observed in AHFpEF cohorts is consistent with the central role of pulmonary vascular remodelling and RV-PA uncoupling in HFpEF pathophysiology [3, 51–57], and sensitivity analyses further supported the robustness of the observed associations. However, the absence of a significant interaction (*p* = 0.64) and the lack of LVEF-stratified estimates in mixed-LVEF cohorts preclude definitive conclusions.

Importantly, RV-PA coupling in AHF is a dynamic parameter and the prognostic relevance of RV-PA coupling probably extends beyond a single baseline assessment [26, 46, 58], because serial measurement may gauge decongestion adequacy alongside weight loss and natriuresis [26, 46, 58]. In Anastasiou et al. [26], persistent uncoupling at discharge (30.8% of 490 patients) independently predicted death or HF rehospitalization, whereas uncoupling that normalized carried no excess risk. It is the failure to restore coupling, not initial uncoupling, that identifies the long-term high-risk phenotype.

Pharmacotherapy may act on both terms of the RV-PA ratio. In a meta-analysis of eight studies, sodium-glucose cotransporter-2 inhibitors (SGLT2i) were associated with higher TAPSE (MD 1.47; 95% CI 1.01–1.93; *p* < 0.01), lower PASP (MD − 5.23; 95% CI − 7.81 to − 2.66; *p* < 0.01), as well as with higher RV fractional area change (MD 5.52; 95% CI 4.23–6.82; *p* < 0.01) during follow-up, suggesting that SGLT2i may improve RV-PA coupling [59]. Rhythm may act in the same direction, as restoration of sinus rhythm has been shown to improve RV-PA coupling [60] - the convalescent state therefore reflects more than decongestion alone.

### LV-Ao coupling and early in-hospital outcomes

AHF is a dynamic syndrome in which an acute precipitant acts on a vulnerable cardiovascular substrate [2]. Within this setting, LV-Ao coupling reflects the immediate interaction between ventricular contractility and arterial load [61, 62].

While LV-Ao coupling has been associated with long-term outcomes in chronic HFrEF [63], our meta-analysis supports its prognostic relevance in AHF through an independent association only with adverse in-hospital cardiovascular outcomes. Among the studies evaluating LV-Ao coupling, only two reported multivariable-adjusted estimates using comparable effect measures, and both assessed in-hospital events [42, 43]. One evaluated a composite of death, invasive ventilation, intra-aortic balloon pump, and renal replacement therapy (adjusted for comorbidities) [43], whereas the other evaluated in-hospital mortality (adjusted for age, sex, Mayo Cardiac Intensive Care Unit Admission Risk Score, Charlson Comorbidity Index, and number of vasoactive drugs) [42]. Despite differences in endpoint definition and multivariable adjustment, both demonstrated an independent association between LV-Ao uncoupling and early adverse clinical outcomes.

LV-Ao uncoupling, assessed with non-invasive measures, is notoriously difficult to track during serial examinations in the same patient. LV pressure-strain analysis, as a surrogate for true LV myocardial work, is prognostically relevant [48, 49] but does not capture the full complexity of information provided by true VAC. Nonetheless, bedside implementation of this approach during stress testing has been described in previous work from our group [64] and is feasible.

### Clinical implications

Two decades ago, Cotter et al. demonstrated that the interaction between cardiac power and systemic vascular resistance defines the hemodynamic phenotype of AHF, a concept subsequently framed by Tan et al. within ventriculo-arterial function curves [65, 66].

Our findings support the use of simple, bedside, non-invasive measures, particularly TAPSE/PASP, as prognostic tools in AHF. The consistency of the available evidence supports a threshold of approximately 0.36–0.38 mm/mmHg (the most common cut-off across included Western cohorts, validated in our sensitivity analysis, Supplemental Fig. 3) as a practical risk stratification criterion at or within 24 h of admission [30, 67]. Beyond admission assessment, serial evaluation of RV-PA coupling may help monitor treatment response and identify patients with persistent hemodynamic vulnerability [68]. Furthermore, restoration of normal RV-PA coupling with medical therapy may serve as a marker of eligibility for interventional treatments such as transcatheter edge-to-edge repair of severe tricuspid or mitral regurgitation [26, 69].

VAC surrogates may be positioned alongside established risk scores. Adding TAPSE/PASP to the Emergency Heart Failure Mortality Risk Grade improved prognostic evaluation in AHF [30]. A risk stratification gap is also apparent beyond the acute setting. In elderly recipients of an implantable cardioverter-defibrillator for HFrEF, the MADIT-II, FADES, PACE and SHOCKED scores rely on demographic, rhythm-related and renal variables, and none incorporates a measure of RV function or VAC [70]. Therefore, VAC surrogates may help to refine rather than duplicate existing stratification.

Finally, non-invasive assessment of LV-Ao coupling complements standard hemodynamic monitoring in high-risk AHF. Previous work by Mihaileanu, Antohi, and colleagues highlighted that VAC provides physiological information beyond LVEF [22, 23]. The modest relationship between LVEF and Ees further underscores this distinction (R^2^ = 0.27) [22]. Accordingly, in patients where escalation to vasoactive or mechanical support is being considered, coupling quantification provides a physiologically grounded framework for distinguishing afterload-predominant uncoupling from contractility-predominant uncoupling, beyond what blood pressure or LVEF alone can determine [22, 23, 61, 71].

### Future research: standardization and the emerging role of artificial intelligence

The lack of standardized cut-off values remains a major barrier to the clinical implementation of VAC. The variability in TAPSE/PASP thresholds observed across studies underscores the need for prospective multicentre validation. Artificial intelligence may facilitate more consistent assessment of VAC, although its role in routine clinical practice remains to be established. A comparable trajectory is already apparent in atrial fibrillation, where artificial intelligence now spans detection, risk stratification, imaging and rhythm-control decision-making [72]. Ultimately, prospective randomized studies are needed to determine whether VAC-guided therapeutic strategies improve clinical outcomes [73–75].

### Limitations

*Our study has several limitations*. First, most included studies were observational, and although only multivariable-adjusted estimates were pooled, residual confounding cannot be excluded. Second, definitions and cut-off values for ventriculo-arterial uncoupling varied across studies. However, sensitivity analyses restricted to studies using TAPSE/PASP cut-off values between 0.36 and 0.38 mm/mmHg, excluding those applying a higher threshold, yielded consistent results, with preserved direction and magnitude of effect. Meta-regression analysis further demonstrated that the study-level TAPSE/PASP cut-off (ten cohorts) did not significantly explain heterogeneity (*p* = 0.41; pseudo-R² = 22%; Supplemental Fig. 5). Third, there was substantial variability in follow-up duration and in the distribution of AHF cohorts according to LVEF. To mitigate these sources of heterogeneity, we performed sub-analyses stratified by medium (12 months) and long-term follow-up for all-cause mortality, as well as subgroup analyses according to LVEF. Fourth, LV-Ao coupling was assessed in relatively few studies, limiting the strength of conclusions regarding long-term outcomes. Moreover, the pooled estimate for in-hospital adverse cardiovascular events combined non-identical study-level composites (in-hospital mortality in one cohort; a composite of support measures or death in the other). Despite an I² of 0%, this clinical heterogeneity in outcome definition warrants cautious, hypothesis-generating interpretation. Fifth, the funnel-plot analysis of the studies included in the primary outcome consisted of only four studies, raising concerns about the reliability of funnel-plot symmetry, and therefore these results should be interpreted with caution.

Finally, the two non-invasive indices, TAPSE/PASP and Ea/Ees, may be interpreted as surrogates of VAC and may not fully reproduce invasive hemodynamic assessments. In addition, the Chen single-beat method may systematically underestimate Ees, potentially resulting in higher derived Ea/Ees ratio [76].

## Conclusion

In patients hospitalized with AHF, non-invasive measures of VAC provide meaningful prognostic information. RV-PA uncoupling is independently associated with increased all-cause mortality, whereas LV-Ao uncoupling is associated with in-hospital adverse cardiovascular events. These findings underscore the clinical relevance of ventriculo-arterial interaction in AHF and support further investigation of VAC-based indices as tools for risk stratification and potential therapeutic guidance.

## Supplementary Information

Below is the link to the electronic supplementary material.

Supplementary Material 1

## Data Availability

No datasets were generated or analysed during the current study.
