# Supplementary material for: Ventriculo-arterial coupling in acute heart failure: prognostic and therapeutic insights - a systematic review and meta-analysis of multivariable-adjusted data
Source: Heart Fail Rev. 2026 Oct 6;31(1):112. doi: 10.1007/s10741-026-10680-w (PMC13642461; doi:10.1007/s10741-026-10680-w)
Supplement: Supplementary file 1 [file 10741_2026_10680_MOESM1_ESM.docx]

**Supplementary Material**

**Ventriculo-arterial coupling in acute heart failure: prognostic and therapeutic insights – a systematic review and meta-analysis of multivariable adjusted data**

Dragos Guz^1,2,+, *^, Daniela Filipescu^1,2,+^, Beth Davison^3,4^, Elena-Laura Antohi^1,2^, Andrew P. Ambrosy^5,6,7^, Serban Mihaileanu^8^, Oliviana Geavlete^1,2^, Sean P. Collins^9^, Razvan Radu^1,2^, Marianna Adamo^10^, Nicolas Girerd^11^, Radu Filipescu^1,2^, Luca Monzo^11^, Jan Biegus^12,13^, Gad Cotter^3,4^, Ovidiu Chioncel^1,2^

**List of Contents**

*Supplemental Figure 1.* Leave-one-out sensitivity analysis (all-cause mortality; heart failure hospitalization; composite outcome of all-cause mortality and heart failure hospitalization).

*Supplemental Figure 2.* Analysis of RV–PA uncoupling across the LVEF spectrum with exclusion of the Falsetti study (29) reduced heterogeneity and yielded a statistically significant association with all-cause mortality. LVEF = left ventricular ejection fraction; RV–PA = right ventricle–pulmonary circulation.

*Supplemental Figure 3.* Sensitivity analyses restricted to studies using TAPSE/PASP cut-off values between 0.36 and 0.38 mm/mmHg yielded consistent results with the main analysis, with preserved direction and magnitude of effect.

*Supplemental Figure 4.* Funnel plot analysis of all-cause mortality for the A. 12-month follow-up subgroup B. All available follow-up periods combined.

*Supplemental Figure 5.* Meta-regression of RV–PA uncoupling and all-cause mortality on the TAPSE/PASP cut-off.

*Supplemental Table 1.* Study-level risk-of-bias assessment of the included studies (ROBINS-I domains for non-randomized cohort studies; RoB 2 for the randomized control trial).

*Supplemental Figure 1*. Leave-one-out sensitivity analysis of the *A*. Primary outcome of all-cause mortality. *B*. Secondary outcome of heart failure hospitalization. *C.* Secondary composite outcome of all-cause mortality and heart failure hospitalization.

*Supplemental Figure 2*. Analysis of RV–PA uncoupling across the LVEF spectrum with exclusion of the Falsetti study (29) reduced heterogeneity and yielded a statistically significant association with all-cause mortality. LVEF = left ventricular ejection fraction; RV–PA = right ventricle–pulmonary circulation.

*Supplemental Figure 3*. Sensitivity analyses restricted to studies using TAPSE/PASP cut-off values between 0.36 and 0.38 mm/mmHg yielded consistent results with the main analysis, with preserved direction and magnitude of effect.

*Supplemental Figure 4*. Funnel plot analysis of all-cause mortality for the A. 12-month follow-up subgroup B. All available follow-up periods combined.

Abbreviation: ACM = all cause-mortality.

*Supplemental Figure 5.* Meta-regression of RV–PA uncoupling and all-cause mortality on the TAPSE/PASP cut-off.

*Supplemental Table 1*. Study-level risk-of-bias assessment of the included studies (ROBINS-I domains for non-randomized cohort studies; RoB 2 for the randomized control trial).

| No. | Study | Year | Design assessed | Tool | D1 | D2 | D3 | D4 | D5 | D6 | Overall |
| --- | --- | --- | --- | --- | --- | --- | --- | --- | --- | --- | --- |
| 1 | Berthelot et al. | 2014 | Prospective single-arm pre-post ADHF decongestive therapy; VAC assessed before/after IV diuresis | ROBINS-I | Serious | Low | Moderate | Moderate | Low | Moderate | Serious |
| 2 | Trambaiolo et al. | 2019 | Retrospective single-center STEMI with AHF treated with levosimendan; VAC before/after infusion and outcomes | ROBINS-I | Serious | Low | Moderate | Moderate | Low | Moderate | Serious |
| 3 | Nakagawa et al. | 2020 | Prospective multicenter observational HFpEF inpatient cohort; TAPSE/PASP and clinical outcomes | ROBINS-I | Moderate | Low | Moderate | Moderate | Low | Moderate | Moderate |
| 4 | Jentzer et al. LVSWI | 2020 | Retrospective Mayo CICU database; echocardiographic LVSWI and hospital/1-year mortality | ROBINS-I | Moderate | Low | Moderate | Moderate | Low | Moderate | Moderate |
| 5 | Palazzuoli et al. | 2020 | Prospective ADHF cohort; early echocardiography and 6-month death/rehospitalization | ROBINS-I | Moderate | Low | Moderate | Moderate | Low | Moderate | Moderate |
| 6 | Santas et al. | 2020 | Prospective HFpEF AHF cohort; RV dysfunction staging using TAPSE/PASP and TR | ROBINS-I | Moderate | Low | Serious | Moderate | Low | Moderate | Serious |
| 7 | Jentzer et al. TASV/RVSP | 2021 | Retrospective Mayo CICU database; TASV/RVSP and hospital/1-year mortality | ROBINS-I | Moderate | Low | Moderate | Moderate | Low | Moderate | Moderate |
| 8 | Sahiti et al. | 2021 | Prospective single-center AHF cohort with paired echocardiography; myocardial work and 6-month outcomes | ROBINS-I | Moderate | Low | Moderate | Moderate | Low | Moderate | Moderate |
| 9 | Santas et al. | 2021 | Large AHF cohort; advanced right heart dysfunction and recurrent readmissions by LVEF | ROBINS-I | Moderate | Low | Serious | Moderate | Low | Moderate | Serious |
| 10 | Berrill et al. | 2022 | Prospective AHF cohort; RV dysfunction parameters and 2-year mortality | ROBINS-I | Moderate | Low | Moderate | Moderate | Low | Moderate | Moderate |
| 11 | Falsetti et al. | 2022 | Observational ED/AHF cohort; incremental prognostic value of TAPSE/PASP added to EHMRG | ROBINS-I | Moderate | Low | Moderate | Moderate | Low | Moderate | Moderate |
| 12 | Hullin et al. | 2022 | Prospective single-center AHF cohort; decongestion-related change in RV-PA coupling and 1-year outcome | ROBINS-I | Moderate | Low | Moderate | Moderate | Low | Moderate | Moderate |
| 13 | Jentzer et al. LVSWI shock | 2022 | Retrospective Mayo CICU cohort; LVSWI and SCAI shock mortality stratification | ROBINS-I | Moderate | Low | Moderate | Moderate | Low | Moderate | Moderate |
| 14 | Naseem et al. | 2022 | Single-center AHF/HFrEF cohort; TAPSE/PASP and in-hospital mortality | ROBINS-I | Moderate | Low | Moderate | Moderate | Low | Moderate | Moderate |
| 15 | Trambaiolo et al. | 2022 | Prospective single-center ICCU cohort; VAC and in-hospital composite clinical outcomes | ROBINS-I | Moderate | Low | Moderate | Moderate | Low | Moderate | Moderate |
| 16 | Bok et al. | 2023 | Observational AHF cohort; RV-PA coupling and mortality | ROBINS-I | Moderate | Low | Moderate | Moderate | Low | Moderate | Moderate |
| 17 | Bonelli et al. | 2023 | Retrospective single-center AHF cohort; admission TAPSE/PASP and 1-year death/HF hospitalization | ROBINS-I | Moderate | Low | Moderate | Moderate | Low | Moderate | Moderate |
| 18 | Jia et al. | 2023 | Prospective acute HFpEF with CAD cohort; RV-arterial uncoupling and composite endpoint | ROBINS-I | Moderate | Low | Moderate | Moderate | Low | Moderate | Moderate |
| 19 | Palazzuoli et al. | 2023 | Multicenter observational AHF cohort; RV function/PA coupling according to LVEF phenotype | ROBINS-I | Moderate | Low | Moderate | Moderate | Low | Moderate | Moderate |
| 20 | Vella et al. | 2023 | Prospective AHF cohort; right heart–renal congestion phenotype and clinical outcomes | ROBINS-I | Moderate | Low | Moderate | Moderate | Low | Moderate | Moderate |
| 21 | Baldetti et al. | 2024 | Observational ADHF cardiogenic shock cohort with invasive hemodynamics; PaE/PAPi and in-hospital death | ROBINS-I | Moderate | Low | Moderate | Moderate | Low | Moderate | Moderate |
| 22 | Berrill et al. | 2024 | Conference abstract/subanalysis; TAPSE/SPAP and 5-year mortality after AHF | ROBINS-I | Serious | Low | Serious | Moderate | Low | Serious | Serious |
| 23 | Bowcock et al. | 2024 | Retrospective NEDA ICU substudy; TAPSE/TRV and survival in critically ill patients | ROBINS-I | Moderate | Low | Moderate | Moderate | Low | Moderate | Moderate |
| 24 | Fauvel et al. | 2024 | Multicenter ICCU/AHF cohort; TAPSE/sPAP and in-hospital prognosis | ROBINS-I | Moderate | Low | Moderate | Moderate | Low | Moderate | Moderate |
| 25 | Liang et al. | 2024 | Prospective multicenter open-label randomized clinical trial after MI with HFrEF; dapagliflozin + sacubitril/valsartan vs sacubitril/valsartan | RoB 2 | Some concerns | Some concerns | Some concerns | Low | Some concerns | Not applicable | Some concerns |
| 26 | Park et al. | 2024 | Large AHF registry; RVGLS/PASP and all-cause mortality across HF phenotypes | ROBINS-I | Moderate | Low | Moderate | Moderate | Low | Moderate | Moderate |
| 27 | Antit et al. | 2025 | Prospective single-center AHF cohort; TAPSE/PASP and mortality/rehospitalization | ROBINS-I | Moderate | Low | Moderate | Moderate | Low | Moderate | Moderate |
| 28 | Burgos et al. | 2025 | Retrospective analysis of prospective AHF registry; HFpEF/HFmrEF and long-term mortality | ROBINS-I | Moderate | Low | Moderate | Moderate | Low | Moderate | Moderate |
| 29 | Decotto et al. | 2025 | Prospective elderly HFpEF AHF cohort; TAPSE/PASP and 1-year all-cause mortality/HF rehospitalization | ROBINS-I | Moderate | Low | Moderate | Moderate | Low | Moderate | Moderate |
| 30 | Fazzini et al. | 2025 | Retrospective Mayo CICU database; LVESV/SV VAC ratio and hospital/1-year mortality | ROBINS-I | Moderate | Low | Moderate | Moderate | Low | Moderate | Moderate |
| 31 | Husain-Syed et al. | 2025 | Multicenter cardiorenal/AHF cohort; renal venous flow changes and liberation from RRT | ROBINS-I | Serious | Low | Moderate | Moderate | Low | Moderate | Serious |
| 32 | Kosmidis et al. | 2025 | Prospective cohort subanalysis/correspondence; RV-PC parameters, inotropic use, and outcomes | ROBINS-I | Serious | Low | Moderate | Moderate | Low | Serious | Serious |
| 33 | De Matteis et al. | 2025 | Retrospective single-center older HFpEF AHF cohort; TAPSE/PASP and in-hospital outcomes/readmission | ROBINS-I | Moderate | Low | Moderate | Moderate | Low | Moderate | Moderate |
| 34 | Padkins et al. | 2025 | Retrospective Mayo CICU cohort; pulmonary effective arterial elastance/RVSP-SBP ratio and mortality | ROBINS-I | Moderate | Low | Moderate | Moderate | Low | Moderate | Moderate |
| 35 | Palazzuoli et al. | 2025 | Multicenter observational AHF cohort; RV-PA uncoupling/strain and 180-day death/HF hospitalization | ROBINS-I | Moderate | Low | Moderate | Moderate | Low | Moderate | Moderate |
| 36 | Anastasiou et al. | 2026 | Consecutive hospitalized AHF cohort; dynamic TAPSE/PASP on admission/discharge and 1-year outcomes | ROBINS-I | Moderate | Low | Moderate | Moderate | Low | Moderate | Moderate |
